# Deaths from infectious respiratory diseases in the Indigenous population: spatial and time series analysis, Minas Gerais, 2000–2023

**DOI:** 10.1590/S2237-96222026v35e20250565.en

**Published:** 2026-04-20

**Authors:** Paulo Filipe Silva Siqueira, Daví Barbosa Pereira de Sousa, Ana Flávia Gamarano Moreira, Brian dos Santos Marchi, Guilherme da Costa Teixeira, Patrícia Ferraz Martins, Waneska Alexandra Alves

**Affiliations:** 1 Universidade Federal de Juiz de Fora, Governador Valadares, MG, Brazil; 2 Universidade Federal de Lavras, Lavras, MG, Brazil; 3 Prefeitura Municipal de Governador Valadares, Secretaria Municipal de Saúde, Governador Valadares, MG, Brazil; 4 Universidade Federal de Ouro Preto, Ouro Preto, MG, Brazil

**Keywords:** Respiratory Tract Infections, Mortality, Indigenous Peoples, Health Information Systems, Time Series Studies, Infecciones del Sistema Respiratorio, Mortalidad, Pueblos Indígenas, Sistema de Información en Salud, Estudios de Series Temporales

## Abstract

**Objective:**

To analyze the temporal trend and spatial distribution of deaths from respiratory tract infections in the Indigenous population residing in Minas Gerais, Brazil, between 2000 and 2023.

**Methods:**

**Methods:**

Time-series and multiple-group analysis of deaths recorded in the Brazilian Mortality Information System (Sistema de Informação sobre Mortalidade, SIM), coded according to the underlying cause of death as respiratory tract infections based on the International Classification of Diseases (ICD). The number of deaths was calculated according to demographic and clinical characteristics. In the trend analysis, the Joinpoint regression model was used to estimate the annual percent change (APC), and for spatial analysis, the global and local Moran’s I indices were applied.

**Results:**

A total of 271 deaths were recorded, predominantly affecting females (51.7%), widowed individuals (30.3%), those with low educational level (49.1%), and older adults (62.7%). In 65.3% of cases, the infection affected the lower respiratory tract, and in 53.5% of cases, the etiologic agent was unspecified. Health macroregions exhibited a downward trend in deaths between 2020 and 2023, namely Central (APC -6.1), East (APC -38.3), and North (APC -25.0). Spatial analysis using Moran’s quadrants identified significant clusters of deaths in São João das Missões, Belo Horizonte, and Bertópolis.

**Conclusion:**

The results demonstrated a high number of deaths among older adults, with marked temporal and territorial inequalities. Specific and sustained public policies are needed to prevent health inequities and ensure equitable access to health services for Indigenous Peoples, alongside improvements to health information systems.

## Resumen

**Objetivo:**

Analizar la tendencia temporal y la distribución espacial de las muertes por enfermedades respiratorias infecciosas en la población indígena residente en Minas Gerais, Brasil, entre 2000 y 2023.

**Métodos:**

Análisis de series temporales y de múltiples grupos de las muertes registradas en el Sistema de Información sobre Mortalidad Brasileño (Sistema de Informação sobre Mortalidade, SIM, codificadas según la causa básica de muerte como enfermedades respiratorias infecciosas de acuerdo con la Clasificación Internacional de Enfermedades (CIE). El número de muertes se calculó según las características demográficas y clínicas. En el análisis de tendencia se utilizó el modelo de regresión por puntos de inflexión (Joinpoint regression), estimándose la variación porcentual anual (VPA); para el análisis espacial se empleó el I de Moran global y local.

**Resultados:**

Se registraron 271 muertes, que afectaron principalmente a personas de sexo femenino (51,7 %), viudas (30,3 %), con bajo nivel educativo (49,1 %) y personas mayores (62,7 %). En el 65,3 % de los casos, la infección afectó el tracto respiratorio inferior y, en el 53,5 %, el agente etiológico no se especificó. Las macrorregiones de salud mostraron un patrón de disminución de las muertes entre 2020 y 2023, entre ellas Centro (VPA -6,1), Leste (VPA -38,3) y Norte (VPA -25,0). El análisis espacial por cuadrantes de Moran evidenció conglomerados significativos de muertes en São João das Missões, Belo Horizonte y Bertópolis.

**Conclusión:**

Los resultados evidenciaron un elevado número de muertes entre personas mayores, con marcadas desigualdades temporales y territoriales. Se requieren políticas públicas específicas y permanentes orientadas a la prevención y al acceso equitativo a los servicios de salud para los pueblos originarios, así como a la mejora de los sistemas de información en salud.

**Ethical aspects**

This research used public domain anonymized databases.

## Introduction 

Infectious respiratory diseases constitute an important group of health conditions and are among the leading infectious causes of death worldwide. They are primarily transmitted through droplets and contact with secretions, and are caused by different infectious agents, mainly viruses, bacteria, and fungi, affecting both the upper and lower respiratory tracts. 

Respiratory infections ranked fourth among the leading causes of death globally in 2023, according to a study (1). In 2021, approximately 344 million new episodes of lower respiratory tract infections occurred worldwide, resulting in 2.5 million deaths (2). A recent study conducted in Brazil showed that between 2009 and 2021 there were approximately 3.5 million cases of acute respiratory infections, with a predominant profile of being male (53.5%), of White race/ethnicity (43.5%), aged over 60 years (41.4%), unvaccinated (35.0%), and without a history of travel (24.3%) (3).

The Brazilian Indigenous population comprises hundreds of ethnic groups with their own cultures, languages, and social organizations. In 2022, they numbered 1,693,535 individuals, corresponding to 0.83% of the national population. In Minas Gerais, approximately 37,000 Indigenous people live in contexts marked by social and environmental inequalities. The average household density (4.6 inhabitants per household) is higher than the national average (3.6), which may increase the transmission of respiratory tract infections in overcrowded settings with inadequate ventilation (4). This scenario is compounded by the precarious infrastructure of health services in Indigenous territories, including shortages of supplies and equipment, lack of access to potable water and waste collection, dependence on air transfers to reach services, limited epidemiological surveillance actions with delays in reporting, and discontinuity of care due to high turnover of health professionals (5,6).

The COVID-19 pandemic, from 2020 to 2022, resulted in 693,853 registered deaths in Brazil, placing the country among the most affected worldwide. Among Indigenous Peoples, 958 deaths were recorded (7). In Minas Gerais, the general population registered 64,447 COVID-19 deaths during the same period. In the Special Indigenous Health District (*Distrito Sanitário Especial Indígena*, DSEI) of Minas Gerais and Espírito Santo, 13 deaths were recorded, highlighting significant regional variations in the pandemic’s impact (8).

Although there are studies on Indigenous health and respiratory diseases in Brazil, most analyses focus on the COVID-19 pandemic period or on national and regional settings. These studies generally do not examine long time series or detail the spatial distribution of registered deaths, thereby limiting understanding of local patterns. In particular, no published analyses combine an extensive historical series with a spatial approach focused on the Indigenous population of Minas Gerais.

Therefore, a gap persists regarding the historical and territorial behavior of respiratory tract infections among Indigenous Peoples in Minas Gerais. This study aims to fill this gap by investigating the temporal trend (2000–2023) and spatial distribution of deaths from respiratory tract infections among Indigenous residents in municipalities of Minas Gerais, providing evidence useful for culturally appropriate planning and policies.

## Methods 

### Study design

This is a time series, multiple-group study of deaths recorded in the Brazilian Mortality Information System (*Sistema de Informação sobre Mortalidade*, SIM), coded by underlying cause of death as respiratory tract infections, among the Indigenous population in Minas Gerais between 2000 and 2023 (9). The units of analysis were macroregions for temporal analysis and municipalities for spatial distribution.

### Study setting

Minas Gerais has 853 municipalities, making it the largest federation unit by number of municipalities. It has the second-largest population in the country, approximately 20.5 million inhabitants in 2022, of which 0.18% self-identify as Indigenous (4). 

### Data sources

Secondary data from SIM were used, made available by the Department of Informatics of the Brazilian Unified Health System Information Technology Department of the Ministry of Health (*Departamento de Informática do Sistema Único de Saúde do Ministério da Saúde*, DATASUS/MS), accessed via TabWin (through file transfer). The databases were extracted between 20 and 31 October 2024.

### Participants 

According to Ministry of Health guidelines, the death certificate is a mandatory document, and the “race/skin color” variable does not permit the “ignored” option. The responsible person must be asked to provide the most accurate race/skin color of the deceased (White, Black, Asian, Brown [Brazilian mixed race], and Indigenous) (10). 

Records in which the race/skin color variable was filled as Indigenous, and the underlying cause of death was coded, according to the International Statistical Classification of Diseases and Related Health Problems 10th Revision (ICD-10), as belonging to the respiratory infection group were included (9,11). Deaths from causes unrelated to infectious respiratory diseases, as well as individuals who were nonindigenous and/or nonresidents of Minas Gerais, were excluded.

### Variables 

For this study, the demographic and clinical variables were analyzed descriptively. The sociodemographic variables were: sex (male, female), marital status (married, divorced, single, stable union, widowed, unknown/missing), educational level (in years of study: 1–3, 4–7, 8–11, ≥12, none, unknown/missing), age group (in years: 0–19, 20–39, 40–59, ≥60), and health macroregion of residence (Central, Central-South, Jequitinhonha, East, East-South, Northeast, Northwest, North, West, Southeast, South, Triângulo do Norte, Triângulo do Sul, Vale do Aço). The clinical variables were: etiologic agent (bacteria, virus, fungus, parasites, unspecified) and anatomical site of infection (upper respiratory tract, lower respiratory tract, systemic) (10) (Supplementary Table 1).

The outcomes analyzed were the absolute number of deaths (n) and the proportion (%). For proportional mortality, numbers were calculated by demographic characteristics (sex, age group, educational level, marital status) and clinical characteristics (etiology, anatomical site of infection).

### Bias 

To reduce potential selection and information biases in record selection, only records in which the race/skin color was clearly indicated as Indigenous, and the underlying cause of death was selected and established according to ICD-10, were included. Codes with inconclusive infectious etiology were excluded from the analysis. Furthermore, to ensure that deaths corresponded to Indigenous individuals residing in Minas Gerais, only localities with specific municipality codes for the state were included. 

### Data analysis

The proportion of deaths was calculated for age group, sex, marital status, and educational level by dividing the number of deaths in each category by the total number of deaths and multiplying by 100. The same procedure was applied for proportional deaths, considering two variables simultaneously (underlying cause and sex; underlying cause and age group). 

For temporal analyses, the sum of deaths was calculated by year of occurrence for each health macroregion. By default, segmented regression imputes a value of 0.50 for years with no recorded deaths, thereby allowing the analysis to proceed.

For spatial analyses, data were grouped into the periods 2000–2009, 2010–2019, and 2020–2023, due to the low frequency of deaths in some regions. Deaths were summed by municipality and assigned a value of 0 to municipalities with no records for each period to produce more stable and interpretable estimates. Analyses were performed using the absolute number of deaths per municipality in each period.

### Statistical methods

For time-series analysis, the JoinPoint Regression Program (version 5.3.0) was used to identify inflection points in trends by applying a logarithmic transformation to the data. The model was fitted using a Poisson variance, with the dependent variable () being the number of deaths and the study years as the independent variable (). Additionally, the health macroregions were included as covariates, enabling a more detailed analysis of regional variations (12). The optimal number of inflection points, with a maximum of four tested, was determined using the weighted Bayesian information criterion (WBIC), which evaluates model fit relative to the number of observations in each segment. To characterize trend periods, the annual percent change (APC) and average annual percent change (AAPC) were calculated with 95% confidence intervals (95%CI). Using the logarithmic scale, the APC is obtained as , where is the slope of the segmented regression line fitted to the logarithm of the number of deaths. The AAPC is determined by , where represents the regression segments and is the weight of each segment , proportional to the number of years the segment covers. When no segments are present . Each inflection point indicates a possible change in the trajectory of the series (13).

For the spatial analysis of deaths, a quintile map (five quantiles) was initially created to visualize the distribution of deaths across the state. Next, Moran’s indices were calculated at global and local levels. At the global level, the statistic measures the difference between observed values and the overall mean, resulting in an index ranging from [-1, 1] (14). 

Upon rejection of the hypothesis of spatial randomness, the local indicator of spatial autocorrelation (LISA) was applied to identify municipalities clustered in different Moran quadrants, namely: high-high, representing municipalities with high death values surrounded by neighbors also with high values; low-low, representing the inverse pattern; high-low and low-high, indicating municipalities whose values differ significantly from neighbors, considered as spatial discrepancies (15).

In descriptive analyses, proportions for categorical variables were calculated, and Pearson’s chi-square test was used to assess differences between categories. Additionally, Fisher’s exact test was used to compare proportions, as some cells had fewer than five observations. Tests were conducted at a 0.05 significance level (5.0%). Except for the temporal analysis, all other steps were performed using RStudio (version 2024.12.0-467). 

## Results 

A total of 271 deaths from respiratory tract infections were recorded in Minas Gerais. Females predominated (51.7%), as did individuals aged 60 years or older (62.7%) and children aged 1–3 years (17.7%). Higher incompleteness was observed for educational level, marital status, and etiologic agent. It is noteworthy that the high proportion of missing information for key variables may affect the interpretation of the results (Table 1). The main causes of death were unspecified pneumonias (38.0%), coronavirus infections (25.8%), and unspecified bacterial pneumonia (14.0%). Other relevant causes included acute respiratory failure and unspecified bronchopneumonia (Table 2).

**Table 1:** Absolute (n) and relative (%) frequency of deaths from respiratory tract infections in the Indigenous population, according to demographic and clinical variables. Minas Gerais, 2000–2023 (n=271)

Variables	Deaths n (%)	p-value^a^
Sex		
Female	140 (51.7)	0.63
Male	131 (48.3)	
**Marital status**		
Married	56 (20.7)	<0.001
Divorced	12 (4.4)	
Single	46 (17.0)	
Stable union	6 (2.2)	
Widowed	82 (30.3)	
Ignored/blank	69 (25.5)	
**Educational level** (**years of schooling**)		
1–3	48 (17.7)	<0.001
4–7	26 (9.6)	
8–11	10 (3.7)	
≥12	4 (1.5)	
None	85 (31.4)	
Ignored/blank	98 (36.2)	
**Age group** (years)		
0–19	41 (15.1)	<0.001
20–39	15 (5.5)	
40–59	45 (16.6)	
≥60	170 (62.7)	
**Etiologic agents**		
Bacteria	53 (19.6)	<0.001
Etiologic agentsirus	73 (26.9)	
Not specified	145 (53.5)	
**Anatomical site**		
Lower respiratory tract	177 (65.3)	<0.001
Upper respiratory tract	4 (1.5)	
Systemic	90 (33.2)	

^a^Pearson’s chi-square.

**Table 2:** Absolute (n) and relative (%) frequency of deaths by underlying cause of death, macroregion of residence, sex, and age group. Minas Gerais, 2000–2023 (n=271)

Variables	Sex			Age group (years)					Total n (%)
	Female n (%)	Male n (%)	p-value^a^	0–19 n (%)	20–39 n (%)	40–59 n (%)	≥60 n (%)	p-value^a^	
**Underlying cause of death**			0.409					<0.001	
Pneumonia, unspecified (J18.9)	59 (42.1)	44 (33.6)		22 (53.7)	4 (26.7)	9 (20.0)	68 (40.0)		103 (38.0)
Coronavirus infection, unspecified site (B34.2)	35 (25.0)	35 (26.7)		2 (4.9)	4 (26.7)	16 (35.6)	48 (28.2)		70 (25.8)
Bacterial pneumonia, unspecified (J15.9)	16 (11.4)	22 (16.8)		6 (14.6)	2 (13.3)	4 (8.9)	26 (15.3)		38 (14.0)
Bronchopneumonia, unspecified (J18.0)	9 (6.4)	8 (6.1)		5 (12.2)	-	2 (4.4)	10 (5.9)		17 (6.3)
Acute respiratory failure (J96.0)	8 (5.7)	12 (9.2)		2 (4.9)	1 (6.7)	8 (17.8)	9 (5.3)		20 (7.4)
Tuberculosis of lung, without mention of bacteriological or histological confirmation (A16.2)	7 (5.0)	2 (1.5)		-	3 (20.0)	4 (8.9)	2 (1.2)		9 (3.3)
Influenza with other respiratory manifestations, virus not identified (J11.8)	2 (1.4)	1 (0.8)		1 (2.4)	-	-	2 (1.2)		3 (1.1)
Lobar pneumonia, unspecified (J18.1)	1 (0.7)	-		-	-	1 (2.2)	-		1 (0.4)
Other bacterial pneumonia (J15.8)	1 (0.7)	2 (1.5)		1 (2.4)	-	-	2 (1.2)		3 (1.1)
Sequelae of respiratory and unspecified tuberculosis (B90.9)	1 (0.7)	-		-	-	-	1 (0.6)		1 (0.4)
Unspecified acute bronchitis (J20.9)	1 (0.7)	-		1 (2.4)	-	-	-		1 (0.4)
Respiratory tuberculosis unspecified, without mention of bacteriological or histological confirmation (A16.9)	-	1 (0.8)		-	-	1 (2.2)	-		1 (0.4)
Unspecified acute lower respiratory infection (J22)	-	1 (0.8)		-	-	-	1 (0.6)		1 (0.4)
Tuberculosis of lung, confirmed by sputum microscopy with or without culture (A15.0)	-	1 (0.8)		-	-	-	1 (0.6)		1 (0.4)
Abscess of lung with pneumonia (J85.1)	-	1 (0.8)		-	1 (6.7)	-	-		1 (0.4)
Acute tonsillitis, unspecified (J03.9)	-	1 (0.8)		1 (2.4)	-	-	-		1 (0.4)
Health macroregion			0.949					<0.001	
Central	33 (23.6)	31 (23.7)		3 (7.3)	4 (26.7)	9 (20.0)	48 (28.2)		64 (23.6)
Central-South	3 (2.1)	2 (1.5)		-	-	2 (4.4)	3 (1.8)		5 (1.8)
Jequitinhonha	2 (1.4)	1 (0.8)		1 (2.4)	-	-	2 (1.2)		3 (1.1)
East	6 (5.0)	5 (3.8)		1 (2.4)	3 (20.0)	2 (4.4)	5 (3.5)		11 (4.5)
East-South	3 (2.1)	2 (1.5)		-	-	2 (4.4)	3 (1.8)		5 (1.8)
Northeast	22 (15.7)	20 (15.3)		26 (63.4)	1 (6.7)	4 (8.9)	11 (6.5)		42 (15.5)
Northwest	2 (1.4)	4 (3.1)		-	-	1 (2.2)	5 (2.9)		6 (2.2)
North	40 (28.6)	34 (26.0)		10 (24.4)	2 (13.3)	13 (28.9)	49 (28.8)		74 (27.3)
West	5 (3.6)	4 (3.1)		-	1 (6.7)	-	8 (4.7)		9 (3.3)
Southeast	7 (5.0)	3 (2.3)		-	-	2 (4.4)	8 (4.7)		10 (3.7)
South	9 (6.4)	12 (9.2)		-	3 (20)	4 (8.9)	14 (8.2)		21 (7.7)
Triângulo do Norte	4 (2.9)	5 (3.8)		-	1 (6.7)	3 (6.7)	5 (2.9)		9 (3.3)
Triângulo do Sul	1 (0.7)	1 (0.8)		-	-	-	2 (1.2)		2 (0.7)
Vale do Aço	3 (2.1)	7 (5.3)		-	-	3 (6.7)	7 (4.1)		10 (3.7)
Total	140 (51.7)	131 (48.3)		15 (5.5)	45 (16.6)	170 (62.7)	170 (62.7)		271 (100.0)

^a^Fisher’s exact test and Monte Carlo simulation.

Unspecified pneumonias and coronavirus infections were the leading causes by age group. Among those aged ≥60 years, pneumonias accounted for 40.0% and coronavirus infections for 28.2%. Among individuals aged 40–59 years, coronavirus infections were more frequent (35.6%). A significant association was observed between the underlying cause and age group (p-value<0.001). Pneumonia and coronavirus infections occurred equally in both sexes, with no significant association between cause and sex (p-value>0.050). The highest concentrations of deaths occurred in the macroregions North (27.3%), Central (23.6%), Northeast (15.5%), and South (7.7%), accounting for 74.2% of all deaths (Table 2).

Segmented regression analysis revealed heterogeneous patterns across the macroregions. Periods of marked increase were observed, such as in Central between 2018–2020 (APC 264.8; 95%CI 149.4; 448.8), East between 2017–2019 (APC 113.8; 95%CI 54.3; 185.9), Southeast between 2018–2020 (APC 93.9; 95%CI 49.5; 138.8), South (APC 68.1; 95%CI 27.1; 109.0), and for the state as a whole during 2018–2020 (APC 60.3; 95%CI 31.8; 85.1). Conversely, significant declines were observed in the most recent period in Minas Gerais, 2021–2023 (APC -49.2; 95%CI -64.6; -32.4), as well as in the Central, East, Southeast, and South macroregions, all showing reductions greater than -38% over the same interval. Some regions exhibited predominantly stable patterns, such as Central-South, Jequitinhonha, East-South, West, Triângulo do Norte, and Triângulo do Sul, while the Northwest showed a more moderate upward trend between 2015 and 2023 (APC 18.0; 95%CI 8.8; 53.1). Vale do Aço was the only region to display a consistent downward global trend during the analyzed period (2011–2023) (APC -10.1; 95%CI -33.3; -2.9). In summary, a common pattern of sharp increases in deaths between 2018 and 2020, followed by a marked decline from 2021 onward, was observed, although the intensity of this pattern varied across macroregions. The main divergences were observed in Vale do Aço, with a consistent reduction, and in regions such as Northwest and Northeast, where moderate or stable trends predominated (Table 3).

**Table 3:** Temporal trends, annual percent change (APC), average annual percent change (AAPC), and 95% confidence interval (95%CI) in the number of deaths from infectious respiratory diseases in the Indigenous population, according to health macroregion. Minas Gerais, 2000–2023 (n=271)

Macroregion	Period	Trendsb	Total period
		APC (95%CI)	AAPC (95%CI)
Central	2009–2017^a^	-19.7 (-57.4; -12.2)	7.4 (3.2; 14.6)
	2018–2020^a^	264.8 (149.4; 448.8)	
	2021–2023	-66.1 (-79.0; -54.1)	
Central-South	2000–2023^a^	-	1.1 (0.6; 3.1)
Jequitinhonha	2000–2023^a^		1.4 (-0.3; 3.2)
East	2009–2016^a^	-10.8 (-47.2; -3.7)	3.0 (-0.2; 7.7)
	2017–2019^a^	113.8 (54.3; 185.9)	
	2020–2023	-38.3 (-54.1; -25.6)	
East-South	2000–2023^a^	-	3.0 (0.2; 6.4)
Northeast	2000–2023^a^	-	6.4 (1.9; 13.5)
Northwest	2015–2023^a^	18.0 (8.8; 53.1)	5.9 (2.9; 8.9)
North	2020–2023	-25.0 (-56.7; 3.5)	10.4 (6.9; 17.3)
West	2000–2023^a^	-	4.6 (1.0; 9.6)
Southeast	2006–2008^a^	54.6 (-4.3; 94.0)	-0.7 (-5.0; 2.8)
	2009–2017^a^	-15.0 (-32.1; -10.0)	
	2018–2020^a^	93.9 (49.5; 138.8)	
	2021–2023^a^	-58.0 (-73.4; -41.6)	
South	2002–2017^a^	6.0 (-3.2; 14.4)	-5.0 (-9.1; -1.2)
	2018–2020^a^	68.1 (27.1; 109.0)	
	2021–2023	-58.3 (-75.3; -35.6)	
Triângulo do Norte	2000–2023^a^	-	6.0 (2.5; 10.7)
Triângulo do Sul	2000–2023^a^	-	0.5 (-1.0; 2.1)
Vale do Aço	2011–2023^a^	-10.1 (-33.3; -2.9)	3.3 (-3.8; 13.9)
Minas Gerais	2018–2020	60.3 (31.8; 85.1)	5.0 (1.8; 8.5)
	2021–2023	-49.2 (-64.6; -32.4)	

^a^The series included imputation of the value 0.5 in some years due to the presence of years without Indigenous deaths; ^b^ Positive percent change values, when statistically different from zero (considering the confidence interval), indicate an increasing trend; negative values indicate a decreasing trend; all others were interpreted as a stationary trend.

**Figure 1:**
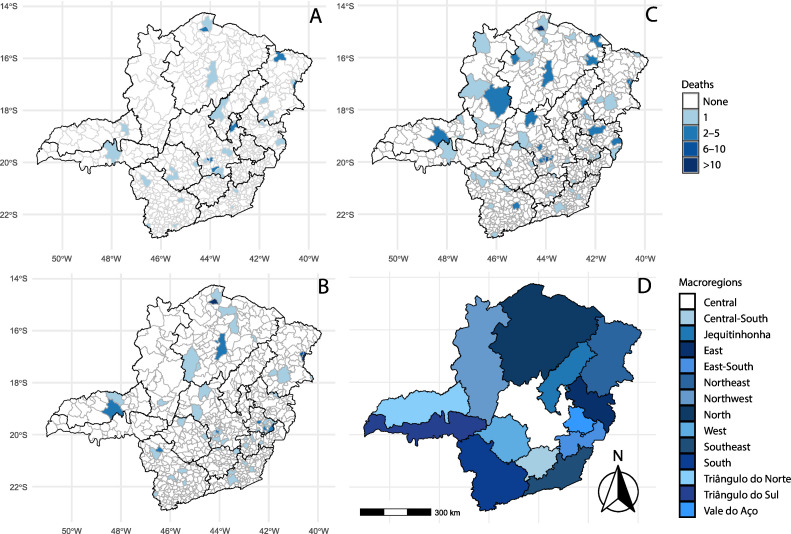
Number of deaths from infectious respiratory diseases between 2000–2009 (A), 2010–2019 (B), 2020–2023 (C), and 2000–2023 by health macroregion (D) in the Indigenous population, according to municipality of residence, spatial distribution using the quantile classification method. Minas Gerais, 2000–2023 (n=271)

**Figure 2:**
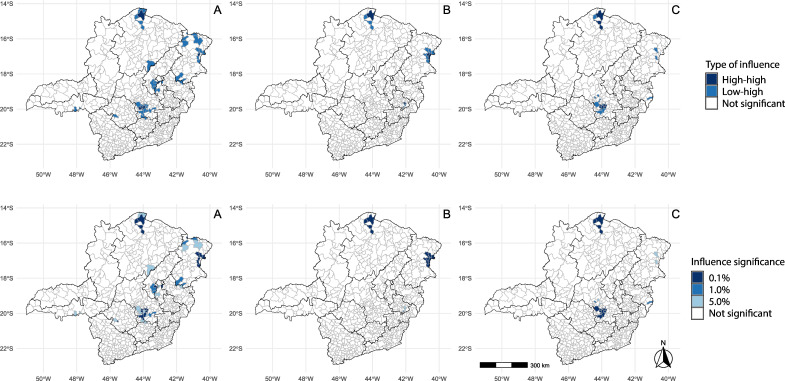
Intermunicipal spatial influence of deaths from infectious respiratory diseases in the Indigenous population between 2000–2009 (A), 2010–2019 (B), and 2020–2023 (C), analysis using local indicators of spatial association (LISA) with Moran’s quadrants. Minas Gerais, 2000–2023 (n=271)

Regarding temporal trend and spatial distribution, deaths increased during 2000–2009 and 2010–2019, followed by a decrease in 2020–2023, although more municipalities recorded deaths in this latter period, maintaining a pattern similar to the second period. In all periods, São João das Missões (North) recorded the highest numbers of deaths: 2000–2009 (n=4), 2010–2019 (n=28), and 2020–2023 (n=17), followed by Bertópolis (Northeast), which also had a high number of deaths in 2010–2019 (n=10) and 2020–2023 (n=3) (Figure 1). The global Moran’s index indicated significant spatial autocorrelation across all three periods: 2000–2009, 0.07; 2010–2019, 0.05; and 2020–2023, 0.09 (p-value<0.010) (Figure 2).

LISA analysis identified significant spatial patterns throughout the series. Between 2000 and 2009, clusters were concentrated in the Central region, notably Belo Horizonte, Betim, Contagem, and Materlândia (high-high), as well as Brumadinho and Ribeirão das Neves (low-high). In the Northeast and North, Santa Helena de Minas and Manga (high-high) and Itacarambi (low-high) stood out. Between 2010 and 2019, some clusters decreased, but critical areas such as Bertópolis and Ubaporanga (high-high) persisted. During 2020–2023, areas of high mortality expanded, particularly in the Central region (Belo Horizonte, Contagem, Santa Luzia, Sabará, Ribeirão das Neves), while patterns persisted in the North (Manga and Itacarambi) and in Brumadinho (low-high) (Figure 2).

## Discussion 

Findings revealed that Indigenous deaths from respiratory tract infections were mostly concentrated among females aged 60 years or older, widowed individuals, and those with low educational levels, with the etiologic agent often unspecified. Deaths were concentrated in the North macroregion, especially São João das Missões, and in the Northeast macroregion, especially in Bertópolis. Higher occurrences were noted during 2000–2009 and 2010–2019, followed by a reduction between 2020–2023.

Therefore, respiratory tract infections represent a public health problem among vulnerable populations such as Indigenous Peoples (2). In this context, the susceptibility of older adults to pneumonia, particularly Indigenous elders, may be related to multiple factors, including immune system decline with aging, the presence of chronic diseases, as well as alterations in respiratory function and swallowing difficulties, which increase the risk of aspiration (1,16,17).

The high frequency of deaths from ill-defined causes without specification of the etiologic agent, such as “unspecified pneumonia,” is a recurring challenge in mortality analyses, as discussed in similar studies (11,17). Difficulty in determining the causative agent was also highlighted in an ecological study on infant mortality from respiratory tract infections in Brazil between 2009 and 2018, in which 94.4% of deaths had unspecified pneumonia as the main cause (11). This pattern may reflect limited access to health services and medical care, deficiencies in laboratory monitoring, and errors in death certificate completion, thereby compromising data quality and hindering understanding of the determinants of morbidity and mortality in this population (10,18).

Spatial patterns indicate significant clusters of deaths, particularly in the North (São João das Missões) and Northeast (Bertópolis). São João das Missões hosts Indigenous residents of Terra Xacriabá (79.8% of the local population), with a high number of death records, which may be attributed to social and political neglect linked to precarious water supply, exacerbated by recurrent droughts, forcing many communities to face a shortage of potable water (19). This may be related to the so-called “lethal triad”: deforestation, wildfires, and respiratory tract infections (20). The constant use of fire in land management, resulting in deforestation to clear new areas for agriculture, may have intensified respiratory tract infections in the region (19,20). 

Spatial patterns suggest that factors such as hospital infrastructure, patient displacement, and socioeconomic differences influence the number of deaths (21). In the North and Northeast macroregions (Itacarambi and Ubaporanga), underreporting or migration of death records may mask local patterns. Structural and regional inequalities particularly affect rural areas (22). Moreover, some macroregions showed stable or unstable temporal patterns, which may result from occasional fluctuations in small absolute numbers, underreporting, errors in death certificates, migration of Indigenous populations, and inequalities in access to health care. These factors complicate the identification of linear trends and require caution in interpreting the results.

The observed temporal distribution aligns with the spatial distribution analyzed, where the pattern of deaths among the Indigenous population was not uniform, reflecting socio-spatial inequalities. Therefore, the significant increase in death records in the North between 2005 and 2019 may reflect environmental impacts from mining, including water contamination and reduced biodiversity (23). Land conflicts and rising violence against Indigenous peoples may have increased the social vulnerability of these populations (24). The significant reduction in records in the Southeast macroregion between 2009–2017 and in Jequitinhonha between 2008–2010 may reflect structural improvements in health care and food security in these regions (25,26).

In the Southeast, expansion of the Family Health Strategy (*Estratégia Saúde da Família*, FHS) and strengthening of primary care may have contributed to the reduction of infectious and preventable diseases, such as the incorporation of community health agents into FHS teams in Indigenous communities (25). In Jequitinhonha, initiatives focused on food security were key to this decline. Social protection and healthy dietary practices reinforced and expanded access to nutritious foods (25,26). 

Between 2019 and 2021, there was a marked increase in deaths among the Indigenous population, especially in the Central region of the state, an area that, due to lower medical infrastructure, received a higher flow of severe COVID-19 patients (27,28). This increase can be attributed not only to individual factors but also to social and environmental determinants. Greater exposure in areas of social vulnerability, combined with precarious housing conditions, high household density, and limited access to health services, significantly increased the risk of transmission and disease severity (28). Therefore, the rapid rise in Indigenous deaths during the pandemic represents a relevant public health milestone for understanding the findings of this study. 

In 2022 and 2023, the reduction in deaths may be attributed to expanded COVID-19 vaccination coverage and Indigenous health surveillance and care actions (1,27). However, fluctuations in regional trends indicate persistent inequalities in access to and quality of health care (27). Macroregions exhibit variation in the temporal trend of mortality, underscoring the need for continuous monitoring and the implementation of health equity policies.

Indigenous populations face structural vulnerabilities that amplify both exposure and severity to respiratory tract infections (19). Precarious access to essential services, such as basic sanitation, potable water, and adequate nutrition, creates unsanitary environments, favors malnutrition, and compromises immunity, increasing susceptibility to infections (16). Geographical and linguistic barriers, overcrowded housing, and institutional bias hinder the implementation of preventive measures and access to qualified medical care, thereby limiting the impact of health interventions (27,29). These structural factors increase both the incidence and number of deaths from respiratory diseases, reinforcing the need for integrated public policies that prioritize primary care, sanitation, nutrition, and health education tailored to the cultural specificities of Indigenous communities (16).

Finally, several important limitations of this study should be noted, including the use of secondary data subject to errors due to racial misclassification, poorly defined causes of death, reliance solely on the underlying cause of death, and underreporting among Indigenous populations in death certificates (30,31). Additionally, heterogeneity in data quality over time, influenced by factors such as changes in death surveillance and infrastructure, may introduce temporal bias in trend analysis (30,31). Census data inconsistencies regarding Indigenous populations and their territories compromise the calculation of specific mortality rates standardized by population at risk.

It is also important to note that the analysis was restricted to the underlying cause of death; standardized rates were not calculated due to the absence of municipal-level Indigenous population data, and the heterogeneity and limitations of the data may affect the robustness of the observed trends. The imputation of minimal values for years without records was necessary to fit the JoinPoint model in a series with long sequences of zeros. However, it is recognized that this strategy may increase trend instability in rare-event series, which is why the interpretation of results was conducted with caution.

Despite these limitations, comparing the results of this study with previous investigations contributes to a better understanding of the dynamics of deaths from respiratory tract infections among Indigenous Peoples in Minas Gerais. 

 It is concluded that the number of deaths from respiratory infections among Indigenous residents of Minas Gerais exhibited an uneven distribution in time and space, with higher concentrations in the North of the state and a more pronounced impact on older adults with low years of schooling, residing in socioeconomically vulnerable areas. The high proportion of unspecified causes highlights weaknesses in both access to qualified care and the proper recording of death information.

In this context, strengthening health care in regions inhabited by Indigenous peoples is recommended, with an emphasis on active surveillance, improving the reporting of underlying causes of death through the action of Death Surveillance, and addressing preventable causes. Priority strategies include: i) improving training for Indigenous health professionals to complete death certificates correctly; ii) implementing local epidemiological and death surveillance systems to enable near-real-time monitoring; iii) reinforcing primary care with itinerant multiprofessional teams in villages and rural communities; and iv) creating integration protocols among municipalities, the state, and the Special Secretariat for Indigenous Health (*Secretaria Especial de Saúde Indígena*, SESAI) to ensure continuous monitoring of Indigenous health (25,26,32). Given the ethnic-racial focus adopted, it is also essential to improve information systems for Indigenous populations, particularly by ensuring accurate completion of the race/skin color variable and incorporating territorial markers sensitive to Indigenous sociocultural organization (30,32). Such measures can reduce underreporting, improve data quality, and support more effective, targeted public policies.

Evidence further highlights the need for studies that integrate data from the DSEI of Minas Gerais and Espírito Santo with information from other health information systems, such as the Influenza Epidemiological Surveillance Information System (*Sistema de Informação da Vigilância Epidemiológica da Gripe*, SIVEP-Gripe) and the Brazilian Health Information System for Indigenous Peoples in Brazil (*Sistema de Informações da Atenção à Saúde Indígena*, SIASI), using data matching techniques. Such integration can enhance the quality of analyses, enabling more precise identification of factors associated with Indigenous deaths from respiratory tract infections and informing culturally sensitive and territorially directed surveillance and health planning actions.
