# Supplementary material for: Deaths from infectious respiratory diseases in the Indigenous population: spatial and time series analysis, Minas Gerais, 2000–2023
Source: Epidemiol Serv Saude. 2026 Apr 20;35:e20250565. doi: 10.1590/S2237-96222026v35e20250565.en (PMC13101430; doi:10.1590/S2237-96222026v35e20250565.en)
Supplement: Supplementary file 1 [file 2237-9622-ress-35-e20250565-supp01-en.pdf]

Supplementary Table 1. International Classification of Diseases codes related to respiratory tract infections by etiologic agent and anatomical site of infection

	International Classification of Diseases
**Etiologic agent**	
Virus	B03, B25.0, B33.4, B34.2, B34.9, J00, J09-12, J17.1, J20.3-7, J21.0
Bacteria	A15-16, A20.2, A21.2, A22.1, A31.0, A38, A42.0, A43.0, A48.1, A78, B90.9, J02.0, J03.0, J13-15, J16.0, J17.0, J20.0-2, J65
Fungi	B37.1, B38.0-2, B39.0-2, B40.0-2, B41.0, B42.0, B44.0-1, B45.0, B46.0, B59, J17.2
Parasites	B58.3, B67.1, J17.3
Not specified	J39.0-1, J85.0-2, J86.0, J86.9, J01-06, J16.8, J17.8, J18-22, J80, J96.0, U04, U04.9
**Anatomical site of infection**	
Lower respiratory tract	A15-16; A20.2; A42.0; A43.0; A48.1; A78; A21.2; A22.1; A31.0; B25.0; B33.4; B37.1; B38.0-2; B39.0-2; B40.0-2; B41.0; B42.0; B44.0-1; B45.0; B46.0; B58.3; B59; B67.1; B90.9; J12-18; J20-22; J65; J85.0-2; J86.0; J86.9
Upper respiratory tract	A38; B03; J00-06; J09-11; J39.0-1
Systemic	B34.2; B34.9; J80; J96.0
