# Supplementary material for: Deaths from infectious respiratory diseases in the Indigenous population: spatial and time series analysis, Minas Gerais, 2000–2023
Source: Epidemiol Serv Saude. 2026 Apr 20;35:e20250565. doi: 10.1590/S2237-96222026v35e20250565.en (PMC13101430; doi:10.1590/S2237-96222026v35e20250565.en)
Supplement: Translation

# Translation: Óbitos por doenças respiratórias infecciosas na população indígena: análise espacial e de séries temporais, Minas Gerais, 2000-2023

**DOI:** 10.1590/S2237-96222026v35e20250565.pt

**Authors:** Paulo Filipe Silva Siqueira, Daví Barbosa Pereira de Sousa, Ana Flávia Gamarano Moreira, Brian dos Santos Marchi, Guilherme da Costa Teixeira, Patrícia Ferraz Martins, Waneska Alexandra Alves

**Affiliations:** 1 Universidade Federal de Juiz de Fora, Governador Valadares, MG, Brasil; 2 Universidade Federal de Lavras, Lavras, MG, Brasil; 3 Prefeitura Municipal de Governador Valadares, Secretaria Municipal de Saúde, Governador Valadares, MG, Brasil; 4 Universidade Federal de Ouro Preto, Ouro Preto, MG, Brasil

**Keywords:** Infecções Respiratórias, Mortalidade, População Indígena, Sistemas de Informação em Saúde, Estudos de Séries Temporais

## Abstract

**Objetivo:**

Analisar a tendência temporal e a distribuição espacial dos óbitos por doenças respiratórias infecciosas em população indígena residente em Minas Gerais, Brasil, entre 2000 e 2023.

**Métodos:**

Análise de séries temporais e múltiplos grupos dos óbitos registrados no Sistema de Informação sobre Mortalidade, codificados pela causa básica de morte como doenças respiratórias infecciosas pela Classificação Internacional de Doenças. O número de óbitos foi calculado segundo características demográficas e clínicas. Na análise de tendência, utilizou-se o modelo de regressão por pontos de inflexão (Joinpoint regression), calculando-se a variação percentual anual (VPA), e para análise espacial, usou-se Índice de Moran global e local.

**Resultados:**

Foram registrados 271 óbitos, atingindo majoritariamente pessoas do sexo feminino (51,7%), viúvos (30,3%), com baixa escolaridade (49,1%) e idosos (62,7%). Em 65,3% dos casos, a infecção atingiu o trato respiratório inferior, e em 53,5% o agente etiológico não foi especificado. Macrorregiões de saúde exibiram padrão de queda nos óbitos entre 2020 e 2023, dos quais, Centro (VPA -6,1), Leste (VPA -38,3) e Norte (VPA -25,0). A análise espacial por quadrantes de Moran evidenciou aglomerações significativas de óbitos em São João das Missões, Belo Horizonte e Bertópolis.

**Conclusão:**

Os resultados evidenciaram alto número de óbitos entre idosos, com expressivas desigualdades temporais e territoriais. Políticas públicas específicas e permanentes são necessárias, visando prevenção e acesso equitativo aos serviços de saúde para os povos originários, com melhorias nos sistemas de informação de saúde.

**Aspectos éticos**

Esta pesquisa utilizou bancos de dados de domínio público e anonimizados.:

## Introdução 

As doenças respiratórias infecciosas constituem importante grupo de agravos à saúde e estão entre as principais causas infecciosas de morte, globalmente. São transmitidas principalmente por gotículas e contato com secreções, sendo causadas por diferentes agentes infecciosos, principalmente vírus, bactérias e fungos, acometendo as vias aéreas superiores e inferiores. 

As infecções respiratórias ocuparam a quarta posição entre as principais causas de morte no mundo em 2023, de acordo com estudo (1). Em 2021, cerca de 344 milhões de novos episódios de infecções respiratórias inferiores ocorreram no mundo, sendo registrados 2,5 milhões de mortes (2). Um estudo recente, realizado no Brasil, evidenciou a ocorrência, entre 2009 e 2021, de aproximadamente 3,5 milhões de casos de infecções respiratórias agudas e perfil predominante para indivíduos do sexo masculino (53,5%), raça/cor branca (43,5%), mais de 60 anos (41,4%), não vacinados (35,0%) e sem histórico de viagem (24,3%) (3).

A população indígena brasileira é composta por centenas de etnias com culturas, línguas e formas próprias de organização social. Em 2022, somavam 1.693.535 pessoas, correspondendo a 0,83% da população nacional. Em Minas Gerais, aproximadamente 37 mil indígenas vivem em contextos marcados por desigualdades sociais e ambientais. A densidade domiciliar média (4,6 moradores por domicílio) é superior à média nacional (3,6), podendo intensificar a transmissão de doenças respiratórias infecciosas em cenários de superlotação e ventilação precária (4). Somam-se a esse cenário a precariedade da infraestrutura dos serviços de saúde em territórios indígenas, com escassez de insumos, equipamentos e até falta de acesso à água potável e coleta de lixo, além da dependência de remoções aéreas para acesso a serviços, da escassez das ações de vigilância epidemiológica, com atrasos nos registros de agravos, e da descontinuidade do cuidado, associada à alta rotatividade de profissionais (5,6).

A pandemia de covid-19, no período de 2020 a 2022, resultou em 693.853 óbitos registrados no Brasil, colocando o país entre os mais afetados no mundo. Entre os povos indígenas, foram registrados 958 óbitos (7). No estado de Minas Gerais, a população geral apresentou 64.447 óbitos por covid-19 no mesmo período. No Distrito Sanitário Especial Indígena (DSEI) Minas Gerais e Espírito Santo, foram registrados 13 óbitos no período, evidenciando importantes variações regionais no impacto da pandemia (8).

Apesar de existirem estudos sobre saúde indígena e doenças respiratórias no Brasil, grande parte das análises concentra-se no período da pandemia de covid-19 ou em recortes nacionais e regionais. Essas produções, em geral, não exploram séries temporais longas nem detalham a distribuição espacial de óbitos registrados, limitando a compreensão de padrões locais. Em particular, não se identificam análises publicadas que combinem série histórica extensa e abordagem espacial focadas na população indígena de Minas Gerais.

Assim, persiste uma lacuna quanto à análise do comportamento histórico e territorial das doenças respiratórias infecciosas entre povos indígenas de Minas Gerais. Este estudo busca preencher essa lacuna, ao investigar a tendência temporal (2000-2023) e a distribuição espacial de óbitos por doenças respiratórias infecciosas entre indígenas residentes em municípios mineiros, oferecendo evidências úteis para planejamento e políticas culturalmente adequadas.

## Métodos 

### Delineamento do estudo

Trata-se de um estudo de séries temporais e múltiplos grupos dos óbitos registrados no Sistema de Informação sobre Mortalidade (SIM), codificados pela causa básica de morte como doenças respiratórias infecciosas, para a população indígena em Minas Gerais, no período 2000-2023 (9). As unidades de análise foram as macrorregiões para a análise temporal e os municípios para a distribuição espacial.

### Local de estudo

Minas Gerais possui 853 municípios, sendo a maior Unidade da Federação em número de municípios. Possui a segunda maior população do país, aproximadamente 20,5 milhões de habitantes em 2022, dos quais 0,18% se autodeclaram indígenas (4). 

### Fonte de dados

Utilizaram-se dados secundários do SIM, disponibilizados pelo Departamento de Informática do Sistema Único de Saúde do Ministério da Saúde (DATASUS/MS), acessados pelo Tabwin (via transferência de arquivos). Os bancos foram extraídos no período de 20 a 31 de outubro de 2024.

### Participantes 

Conforme orientações do Ministério da Saúde, a declaração de óbito é documento obrigatório e a variável raça/cor da pele não admite a alternativa “ignorada”, sendo necessário perguntar à pessoa responsável por prestar as informações sobre o falecido, que informará a melhor opção de raça/cor da pele (branca, preta, amarela, parda e indígena) (10). 

Foram incluídos os registros cuja variável raça/cor da pele estivesse preenchida como indígena e a causa básica de morte codificada, segundo a Classificação Internacional de Doenças (CID-10), como pertencente ao grupo de infecções respiratórias (9,11). Foram excluídos os registros de óbitos com causas não relacionadas às doenças respiratórias infecciosas, bem como os de indivíduos não indígenas e/ou não residentes em Minas Gerais.

### Variáveis 

Para esta pesquisa, foram analisadas descritivamente as variáveis demográficas e clínicas. As variáveis sociodemográficas foram: sexo (masculino, feminino), estado civil (casado, divorciado, solteiro, união estável, viúvo, ignorado/vazio), escolaridade (em anos de estudo: 1-3, 4-7, 8-11, ≥12, nenhuma, ignorado/vazio), faixa etária (em anos: 0-19, 20-39, 40-59, ≥60), macrorregião de saúde de residência (Centro, Centro Sul, Jequitinhonha, Leste, Leste do Sul, Nordeste, Noroeste, Norte, Oeste, Sudeste, Sul, Triângulo do Norte, Triângulo do Sul, Vale do Aço). As variáveis clínicas foram: agente etiológico (bactéria, vírus, fungo, parasitas, não especificado) e sítio anatômico da infecção (vias aéreas superiores, vias aéreas inferiores, sistêmico) (10) (Tabela Suplementar 1).

Os desfechos analisados foram os números absolutos de óbitos (n) e os proporcionais (%). Para a mortalidade proporcional, os números foram calculados segundo características demográficas (sexo, faixa etária, escolaridade, estado civil) e clínicas (agente etiológico, sítio anatômico da infecção).

### Viés 

Para reduzir potenciais vieses de seleção e informação na seleção de registros, optou-se pelos cuja raça/cor da pele indígena estivesse claramente preenchida e a causa básica de morte selecionada e estabelecida pela CID-10. Códigos com etiologia infecciosa considerada inconclusiva foram excluídos da análise. Ademais, para se garantir que os óbitos correspondessem a indivíduos indígenas residentes em Minas Gerais, foram incluídas apenas localidades com códigos específicos para municípios do estado.

### Análise dos dados

Calculou-se o número de óbitos proporcional para faixa etária, sexo, estado civil e escolaridade, considerando-se a divisão do número de óbitos em cada variável dívida pelo total de óbitos, multiplicada por 100. O mesmo foi realizado para as mortes proporcionais com duas variáveis (causa básica e sexo; causa básica e faixa etária). 

Para as análises temporais, a soma dos óbitos foi calculada por ano de ocorrência para cada macrorregião de saúde. Por padrão, a regressão segmentada imputa o valor 0,50 nos anos sem óbitos registrados, permitindo a realização da análise.

Para as análises espaciais, os dados foram agrupados nos períodos 2000-2009, 2010-2019 e 2020-2023, devido à baixa frequência de óbitos em algumas regiões, somando-se os óbitos por município e atribuindo-se zero aos municípios sem registros durante cada período, de modo a serem garantidas estimativas mais estáveis e interpretáveis. As análises foram realizadas com o número absoluto de óbitos por município em cada período.

### Métodos estatísticos

Na análise de séries temporais, utilizou-se o software JoinPoint Regression Program (versão 5.3.0), que permite identificar pontos de inflexão nas tendências ao longo do tempo, aplicando a transformação logarítmica aos dados. O modelo foi ajustado sob variância Poisson e adotando como variável dependente () o número de óbitos, enquanto os anos do período de estudo foram definidos como variável independente (). Ademais, foram incluídas como covariáveis as macrorregiões de saúde, possibilitando uma análise mais detalhada das variações regionais (12). O número ótimo de pontos de inflexão, com um máximo de quatro testados, foi definido com base no critério do menor critério de informação bayesiano ponderado (WBIC), que pondera a qualidade do ajuste pelo número de observações em cada segmento. Para caracterizar os períodos de tendência, calcularam-se a variação percentual anual (VPA) e a variação percentual anual média (VPAM) com intervalo de confiança de 95% (IC95%). Considerando-se a aplicação da escala logarítmica, a VPA é obtida por , em que representa o coeficiente angular (slope) da reta de regressão segmentada ajustada ao logaritmo do número de óbitos. Já a VPAM é determinada por , onde representa os segmentos da regressão e é o peso de cada segmento , proporcional à quantidade de anos que o segmento cobre. Quando não há segmentos, . Cada ponto de inflexão indica uma possível mudança na trajetória da série (13).

Para a análise espacial dos óbitos, inicialmente foi elaborado um mapa de quintis (cinco quantis) para visualização da distribuição dos dados no estado. Em seguida, calcularam-se os índices de Moran nos níveis global e local. No nível global, a estatística mede a diferença entre os valores observados e a média geral, resultando em um índice que varia no intervalo [-1,1] (14). 

Com a rejeição da hipótese de aleatoriedade espacial, aplicou-se o indicador local de autocorrelação espacial (LISA) para se identificarem municípios agrupados em diferentes quadrantes de Moran, a saber: alto-alto, representando municípios com altos valores de óbitos cercados por vizinhos também com valores elevados; baixo-baixo, com padrão inverso ao anterior; alto-baixo e baixo-alto, que indicam municípios cujos valores diferem significativamente dos vizinhos, sendo considerados como discrepâncias espaciais (15).

Nas análises descritivas, foram calculadas as proporções das variáveis categóricas, aplicando-se o teste qui-quadrado de Pearson para identificar diferenças entre classes. Além disso, ao se compararem proporções entre variáveis, utilizou-se o teste exato de Fisher, uma vez que algumas células apresentavam menos de cinco observações. Os testes foram conduzidos considerando um nível de significância de 0,05 (5,0%). Com exceção da análise temporal, as demais etapas foram realizadas no software RStudio (versão 2024.12.0-467). 

## Resultados 

Foram registrados 271 óbitos por doenças respiratórias infecciosas em Minas Gerais. Predominaram mulheres (51,7%), indivíduos com 60 anos ou mais (62,7%) e crianças de 1 a 3 anos (17,7%). Observou-se maior incompletude em escolaridade, estado civil e agente etiológico. Vale ressaltar que a elevada proporção de informações ausentes em variáveis-chave pode influenciar a interpretação dos resultados (Tabela 1). As principais causas de óbito foram pneumonias não especificadas (38,0%), infecções por coronavírus (25,8%) e pneumonia bacteriana não especificada (14,0%). Outras causas relevantes incluíram insuficiência respiratória aguda e broncopneumonia não especificada (Tabela 2).

**Tabela 1:** Frequência absoluta (n) e relativa (%) do número de óbitos por doenças respiratórias infecciosas em população indígena, segundo variáveis demográficas e clínicas. Minas Gerais, 2000-2023 (n=271)

Variáveis	Óbitos n (%)	p-valor^a^
Sexo		
Feminino	140 (51,7)	0,63
Masculino	131 (48,3)	
**Estado civil**		
Casado	56 (20,7)	<0,001
Divorciado	12 (4,4)	
Solteiro	46 (17,0)	
União estável	6 (2,2)	
Viúvo	82 (30,3)	
Ignorado/vazio	69 (25,5)	
**Escolaridade** (anos)		
1-3	48 (17,7)	<0,001
4-7	26 (9,6)	
8-11	10 (3,7)	
≥12	4 (1,5)	
Nenhuma	85 (31,4)	
Ignorado/vazio	98 (36,2)	
**Faixa etária** (anos)		
0-19	41 (15,1)	<0,001
20-39	15 (5,5)	
40-59	45 (16,6)	
≥60	170 (62,7)	
**Agente etiológico**		
Bactéria	53 (19,6)	<0,001
Vírus	73 (26,9)	
Não especificado	145 (53,5)	
**Sítio anatômico**		
Vias aéreas inferiores	177 (65,3)	<0,001
Vias aéreas superiores	4 (1,5)	
Sistêmico	90 (33,2)	

^a^Qui-quadrado de Pearson.

**Tabela 2:** Frequência absoluta (n) e relativa (%) do número de óbitos por causa básica de morte, macrorregião de saúde de residência do óbito, sexo e faixa etária. Minas Gerais, 2000-2023 (n=271)

Variáveis	Sexo			Faixa etária (anos)					Total n (%)
	Feminino n (%)	Masculino n (%)	p-valor^a^	0-19 n (%)	20-39 n (%)	40-59 n (%)	≥60 n (%)	p-valor^a^	
**Causa básica da morte**			0,409					<0,001	
Pneumonia não especificada (J18.9)	59 (42,1)	44 (33,6)		22 (53,7)	4 (26,7)	9 (20,0)	68 (40,0)		103 (38,0)
Infecção por coronavírus de localização não especificada (B34.2)	35 (25,0)	35 (26,7)		2 (4,9)	4 (26,7)	16 (35,6)	48 (28,2)		70 (25,8)
Pneumonia bacteriana não especificada (J15.9)	16 (11,4)	22 (16,8)		6 (14,6)	2 (13,3)	4 (8,9)	26 (15,3)		38 (14,0)
Broncopneumonia não especificada (J18.0)	9 (6,4)	8 (6,1)		5 (12,2)	-	2 (4,4)	10 (5,9)		17 (6,3)
Insuficiência respiratória aguda (J96.0)	8 (5,7)	12 (9,2)		2 (4,9)	1 (6,7)	8 (17,8)	9 (5,3)		20 (7,4)
Tuberculose pulmonar, sem menção de confirmação bacteriológica ou histológica (A16.2)	7 (5,0)	2 (1,5)		-	3 (20,0)	4 (8,9)	2 (1,2)		9 (3,3)
Influenza (gripe) com outras manifestações respiratórias, devida a vírus não identificado (J11.8)	2 (1,4)	1 (0,8)		1 (2,4)	-	-	2 (1,2)		3 (1,1)
Pneumonia lobar não especificada (J18.1)	1 (0,7)	-		-	-	1 (2,2)	-		1 (0,4)
Outras pneumonias bacterianas (J15.8)	1 (0,7)	2 (1,5)		1 (2,4)	-	-	2 (1,2)		3 (1,1)
Sequelas de tuberculose das vias respiratórias e de órgãos não especificados (B90.9)	1 (0,7)	-		-	-	-	1 (0,6)		1 (0,4)
Bronquite aguda não especificada (J20.9)	1 (0,7)	-		1 (2,4)	-	-	-		1 (0,4)
Tuberculose respiratória, não especificada, sem menção de confirmação bacteriológica ou histológica (A16.9)	-	1 (0,8)		-	-	1 (2,2)	-		1 (0,4)
Infecções agudas não especificada das vias aéreas inferiores (J22)	-	1 (0,8)		-	-	-	1 (0,6)		1 (0,4)
Tuberculose pulmonar, com confirmação por exame microscópico da expectoração, com ou sem cultura (A15.0)	-	1 (0,8)		-	-	-	1 (0,6)		1 (0,4)
Abscesso do pulmão com pneumonia (J85.1)	-	1 (0,8)		-	1 (6,7)	-	-		1 (0,4)
Amigdalite aguda não especificada (J03.9)	-	1 (0,8)		1 (2,4)	-	-	-		1 (0,4)
**Macrorregião de saúde**			0,949					<0,001	
Centro	33 (23,6)	31 (23,7)		3 (7,3)	4 (26,7)	9 (20,0)	48 (28,2)		64 (23,6)
Centro Sul	3 (2,1)	2 (1,5)		-	-	2 (4,4)	3 (1,8)		5 (1,8)
Jequitinhonha	2 (1,4)	1 (0,8)		1 (2,4)	-	-	2 (1,2)		3 (1,1)
Leste	6 (5,0)	5 (3,8)		1 (2,4)	3 (20,0)	2 (4,4)	5 (3,5)		11 (4,5)
Leste do Sul	3 (2,1)	2 (1,5)		-	-	2 (4,4)	3 (1,8)		5 (1,8)
Nordeste	22 (15,7)	20 (15,3)		26 (63,4)	1 (6,7)	4 (8,9)	11 (6,5)		42 (15,5)
Noroeste	2 (1,4)	4 (3,1)		-	-	1 (2,2)	5 (2,9)		6 (2,2)
Norte	40 (28,6)	34 (26,0)		10 (24,4)	2 (13,3)	13 (28,9)	49 (28,8)		74 (27,3)
Oeste	5 (3,6)	4 (3,1)		-	1 (6,7)	-	8 (4,7)		9 (3,3)
Sudeste	7 (5,0)	3 (2,3)		-	-	2 (4,4)	8 (4,7)		10 (3,7)
Sul	9 (6,4)	12 (9,2)		-	3 (20)	4 (8,9)	14 (8,2)		21 (7,7)
Triângulo do Norte	4 (2,9)	5 (3,8)		-	1 (6,7)	3 (6,7)	5 (2,9)		9 (3,3)
Triângulo do Sul	1 (0,7)	1 (0,8)		-	-	-	2 (1,2)		2 (0,7)
Vale do Aço	3 (2,1)	7 (5,3)		-	-	3 (6,7)	7 (4,1)		10 (3,7)
Total	140 (51,7)	131 (48,3)		15 (5,5)	45 (16,6)	170 (62,7)	170 (62,7)		271 (100,0)

^a^ Exato de Fisher e simulação de Monte Carlo.

Pneumonias não especificadas e infecções por coronavírus foram as principais causas por faixa etária. Em ≥60 anos, pneumonias corresponderam a 40,0% e coronavírus a 28,2%. Entre 40-59 anos, infecções por coronavírus foram mais frequentes (35,6%). Houve associação significativa entre causa básica e faixa etária (p-valor<0,001). Pneumonias e infecções por coronavírus predominaram em ambos os sexos, sem associação significativa entre causa e sexo (p-valor>0,050). A maior concentração de óbitos ocorreu nas macrorregiões Norte (27,3%), Centro (23,6%), Nordeste (15,5%) e Sul (7,7%), totalizando 74,2% do total (Tabela 2).

O modelo de regressão segmentada revelou padrões heterogêneos nas macrorregiões. Houve períodos de crescimento expressivo, como no Centro entre 2018-2020 (VPA 264,8; IC95% 149,4; 448,8), Leste entre 2017-2019 (VPA 113,8; IC95% 54,3; 185,9), Sudeste entre 2018-2020 (VPA 93,9; IC95% 49,5; 138,8), Sul (VPA 68,1; IC95% 27,1; 109,0) e no estado como um todo no período de 2018-2020 (VPA 60,3; IC95% 31,8; 85,1). Em contrapartida, quedas significativas foram observadas no período mais recente em Minas Gerais 2021-2023 (VPA -49,2; IC95% -64,6; -32,4), bem como nas macrorregiões Centro, Leste, Sudeste e Sul, todas com reduções acima de -38% no mesmo intervalo. Algumas regiões apresentaram comportamentos predominantemente estacionários, como Centro-Sul, Jequitinhonha, Leste do Sul, Oeste, Triângulo do Norte e Triângulo do Sul, enquanto o Noroeste, entre 2015-2023 (VPA 18,0; IC95% 8,8; 53,1), mostrou tendência de crescimento mais moderada. O Vale do Aço foi a única região a apresentar tendência global decrescente no período analisado entre 2011-2023 (VPA -10,1; IC95% -33,3; -2,9). Em síntese, verificou-se um padrão comum de aumento expressivo de óbitos entre 2018 e 2020, seguido de queda acentuada a partir de 2021, embora com intensidades distintas entre as macrorregiões. As principais divergências residem no Vale do Aço, com redução consistente, e em regiões como Noroeste e Nordeste, onde predominaram tendências de crescimento mais moderado ou estacionário (Tabela 3).

**Tabela 3:** Tendências temporais, variação percentual anual (VPA), variação percentual anual média (VPAM) e intervalo de confiança de 95% (IC95%) do número de óbitos por doenças respiratórias infecciosas em população indígena, segundo a macrorregião de saúde. Minas Gerais, 2000-2023 (n=271)

Macrorregião	Período	Tendências^b^	Período total
		VPA (IC95%)	VPAM (IC95%)
Centro	2009-2017^a^	-19,7 (-57,4; -12,2)	7,4 (3,2; 14,6)
	2018-2020^a^	264,8 (149,4; 448,8)	
	2021-2023	-66,1 (-79,0; -54,1)	
Centro Sul	2000-2023^a^	-	1,1 (0,6; 3,1)
Jequitinhonha	2000-2023^a^		1,4 (-0,3; 3,2)
Leste	2009-2016^a^	-10,8 (-47,2; -3,7)	3,0 (-0,2; 7,7)
	2017-2019^a^	113,8 (54,3; 185,9)	
	2020-2023	-38,3 (-54,1; -25,6)	
Leste do Sul	2000-2023^a^	-	3,0 (0,2; 6,4)
Nordeste	2000-2023^a^	-	6,4 (1,9; 13,5)
Noroeste	2015-2023^a^	18,0 (8,8; 53,1)	5,9 (2,9; 8,9)
Norte	2020-2023	-25,0 (-56,7; 3,5)	10,4 (6,9; 17,3)
Oeste	2000-2023^a^	-	4,6 (1,0; 9,6)
Sudeste	2006-2008^a^	54,6 (-4,3; 94,0)	-0,7 (-5,0; 2,8)
	2009-2017^a^	-15,0 (-32,1; -10,0)	
	2018-2020^a^	93,9 (49,5; 138,8)	
	2021-2023^a^	-58,0 (-73,4; -41,6)	
Sul	2002-2017^a^	6,0 (-3,2; 14,4)	-5,0 (-9,1; -1,2)
	2018-2020^a^	68,1 (27,1; 109,0)	
	2021-2023	-58,3 (-75,3; -35,6)	
Triângulo do Norte	2000-2023^a^	-	6,0 (2,5; 10,7)
Triângulo do Sul	2000-2023^a^	-	0,5 (-1,0; 2,1)
Vale do Aço	2011-2023^a^	-10,1 (-33,3; -2,9)	3,3 (-3,8; 13,9)
Minas Gerais	2018-2020	60,3 (31,8; 85,1)	5,0 (1,8; 8,5)
	2021-2023	-49,2 (-64,6; -32,4)	

^a^Série teve imputação de valor 0,5 em algum dos anos devido à presença de anos sem óbitos indígenas; ^b^Valores positivos de variação percentual, desde que estatisticamente diferentes de zero (considerando o intervalo de confiança), indicam tendência crescente, valores negativos indicam tendência decrescente e os demais foram interpretados como tendência estacionária.

Quanto à tendência temporal e à distribuição espacial, observou-se aumento de óbitos entre 2000-2009 e 2010-2019, seguido de redução entre 2020-2023, embora mais municípios tenham registrado óbitos nesse último período, mantendo padrão semelhante ao segundo período. Em todos os períodos, São João das Missões (Norte) apresentou os maiores números de óbitos entre 2000-2009 (n=4), 2010-2019 (n=28) e 2020-2023 (n=17), seguida por Bertópolis (Nordeste), que também registrou número de mortes expressiva entre 2010-2019 (n=10) e 2020-2023 (n=3) (Figura 1). O índice de Moran global indicou autocorrelação espacial significativa nos três períodos analisados: 2000-2009, 0,07; 2010-2019, 0,05; e 2020-2023, 0,09 (p-valor<0,010) (Figura 2).

**Figura 2:** Influência espacial intermunicipal dos óbitos por doenças respiratórias infecciosas em população indígena entre 2000-2009 (A), 2010-2019 (B), 2020-2023 (C), análise por indicadores locais de associação espacial com quadrantes de Moran. Minas Gerais, 2000-2023 (n=271)

**Figura 1:** Número de óbitos por doenças respiratórias infecciosas entre 2000-2009 (A), 2010-2019 (B), 2020-2023 (C) e 2000-2023 por macrorregião (D) em população indígena, segundo município de residência, distribuição espacial por técnica de quantis. Minas Gerais, 2000-2023 (n=271)

A análise LISA identificou padrões espaciais significativos ao longo de toda a série. Entre 2000-2009, os agrupamentos concentraram-se na região Centro, destacando-se Belo Horizonte, Betim, Contagem e Materlândia (alto-alto), além de Brumadinho e Ribeirão das Neves (baixo-alto). No Nordeste e Norte, Santa Helena de Minas e Manga (alto-alto) e Itacarambi (baixo-alto) se destacaram. Entre 2010-2019, alguns agrupamentos diminuíram, mas áreas críticas como Bertópolis e Ubaporanga (alto-alto) persistiram. No período 2020-2023, houve expansão das áreas de alto número de óbitos, principalmente no Centro (Belo Horizonte, Contagem, Santa Luzia, Sabará, Ribeirão das Neves), mantendo-se padrões no Norte (Manga e Itacarambi) e em Brumadinho (baixo-alto) (Figura 2).

## Discussão 

Os achados revelaram que os óbitos indígenas por doenças respiratórias infecciosas se concentraram, em sua maioria, em mulheres com 60 anos ou mais, viúvas e com baixa escolaridade, cujo agente etiológico não foi especificado. Os óbitos se concentraram nas macrorregiões Norte, especialmente em São João das Missões, e Nordeste, com destaque para Bertópolis. Verificou-se maior ocorrência nos anos de 2000-2009 e 2010-2019, seguida de redução entre 2020-2023.

Dessa forma, as doenças respiratórias se colocam como um problema de saúde pública entre as populações vulneráveis, como os indígenas (2). Nesse contexto, a suscetibilidade da população idosa à pneumonia, especialmente para idosos indígenas, pode estar relacionada a múltiplos fatores, como o declínio do sistema imunológico com o envelhecimento, a presença de doenças crônicas, além de alterações na função respiratória e dificuldades na deglutição, que elevam o risco de aspiração (1,16,17).

A elevada frequência de óbitos por causas mal definidas sem a especificação do agente etiológico como “pneumonia não especificada” é um desafio recorrente na análise do número de mortes, como abordado em estudos semelhantes (11,17). A dificuldade em determinar o agente também foi evidenciada em estudo ecológico sobre mortalidade infantil por doenças respiratórias no Brasil entre 2009-2018, no qual 94,4% dos óbitos tiveram pneumonia não especificada como causa principal (11). Esse padrão pode refletir limitações no acesso aos serviços de saúde, assistência médica, deficiências na vigilância laboratorial e falhas no preenchimento das declarações de óbito, comprometendo a qualidade dos dados e dificultando a compreensão dos diferentes determinantes para a morbimortalidade nessa população (10,18).

Os padrões espaciais indicam aglomerados significativos de óbitos, especialmente no Norte (São João das Missões) e Nordeste (Bertópolis). São João das Missões concentra indígenas da Terra Xacriabá (79,8% da população residente) com alto número de registros de óbitos, que pode ser atribuída à negligência social e política atrelada à precariedade no abastecimento de água, agravado pela recorrente seca, levando muitas comunidades a enfrentarem a escassez de água potável (19). Isso pode estar relacionado com a “tríade fatalmente conectada”: desmatamento, queimadas e doenças respiratórias (20). O uso do fogo constante no manejo da terra, provocando o desmatamento para destinação de novas áreas para cultivo da agricultura, pode consistir em fatores que intensificaram as doenças respiratórias na região (19,20). 

Os padrões espaciais sugerem que fatores como infraestrutura hospitalar, deslocamento de pacientes e diferenças socioeconômicas influenciam no número de mortes (21). Nas macrorregiões Norte e Nordeste (Itacarambi e Ubaporanga), pode haver subnotificação ou migração de registros de óbitos, mascarando padrões locais. As desigualdades estruturais e regionais impactam sobretudo as áreas rurais (22). Além disso, algumas macrorregiões apresentaram comportamento temporal estacionário ou instável, o que pode decorrer de flutuações ocasionais em números absolutos reduzidos, subnotificação, falhas nas declarações de óbito, migração de populações indígenas e desigualdades no acesso à saúde. Esses fatores dificultam a identificação de tendências lineares e exigem cautela na interpretação dos resultados.

A distribuição temporal observada está alinhada com a distribuição espacial analisada onde o comportamento dos óbitos para a população indígena não foi uniforme, refletindo desigualdades socioespaciais. Assim, o crescimento significativo nos registros de óbitos no Norte, entre 2005-2019, pode refletir os impactos ambientais da mineração, que resultaram na contaminação hídrica e redução da biodiversidade (23). Conflitos fundiários e o aumento da violência contra indígenas podem ter intensificado a vulnerabilidade social dessas populações (24). A redução significativa dos registros nas macrorregiões Sudeste, entre 2009-2017, e Jequitinhonha, entre 2008-2010, podem refletir avanços estruturais na saúde e segurança alimentar dessas regiões (25,26).

No Sudeste, a ampliação da Estratégia Saúde da Família (ESF) e o fortalecimento da atenção básica podem ter contribuído para a redução de doenças infecciosas e evitáveis, a exemplo da incorporação de agentes comunitários de saúde nas equipes da ESF em comunidades indígenas (25). No Jequitinhonha, iniciativas voltadas para a segurança alimentar foram determinantes para essa queda. Proteção social e práticas alimentares saudáveis reforçaram e ampliaram o acesso a alimentos nutritivos (25,26). 

Entre 2019 e 2021, observou-se um aumento expressivo no número de óbitos entre a população indígena, especialmente no Centro do estado, região que, devido à infraestrutura médica, recebeu um fluxo maior de pacientes graves por covid-19 (27,28). Esse crescimento pode ser atribuído não apenas a fatores individuais, mas também a determinantes sociais e ambientais. A maior exposição em áreas de vulnerabilidade social, associada a condições de moradia precárias, alta densidade domiciliar e acesso limitado aos serviços de saúde, ampliou significativamente o risco de transmissão e agravamento da doença (28). Assim, a rápida elevação dos óbitos indígenas, no período da pandemia, constitui um marco sanitário relevante para a compreensão dos achados desta pesquisa. 

Em 2022 e 2023, a redução nos óbitos pode ser atribuída à ampliação da cobertura vacinal contra a covid-19 e às ações de vigilância e assistência à saúde indígena (1,27). Contudo, flutuações nas tendências regionais apontam para desigualdades persistentes no acesso e na qualidade da atenção à saúde (27). As macrorregiões apresentam variações na tendência temporal dos óbitos, sugerindo a necessidade de monitoramento contínuo e implementação de políticas de equidade em saúde.

As populações indígenas enfrentam vulnerabilidades estruturais que amplificam sua exposição e gravidade às doenças respiratórias infecciosas (19). A precariedade no acesso a serviços essenciais, como saneamento básico, água potável e alimentação adequada, cria ambientes insalubres, favorece a desnutrição e compromete a imunidade, aumentando a suscetibilidade às doenças (16). Barreiras geográficas e linguísticas, moradias superlotadas e preconceito institucional dificultam a implementação de medidas preventivas e o acesso a cuidados médicos qualificados, contribuindo para o impacto limitado das intervenções de saúde (27,29). Esses fatores estruturais elevam tanto a incidência quanto o número de mortes por doenças respiratórias, reforçando a necessidade de políticas públicas integradas, que priorizem atenção primária, saneamento, nutrição e educação em saúde adaptadas às especificidades culturais das comunidades indígenas (16).

Por fim, destacam-se limitações relevantes deste estudo, como análise com dados secundários sujeitos a erros devido à heteroclassificação racial, causas de morte mal definidas, uso apenas da causa básica de morte e subnotificação para a população indígena no preenchimento da declaração de óbito (30,31). Ademais, a heterogeneidade na qualidade da informação ao longo do tempo, influenciada por fatores como mudanças na vigilância do óbito e infraestrutura, pode gerar viés temporal na análise de tendências (30,31). A inconsistência de dados censitários sobre a população indígena e seus territórios compromete a construção de taxas de mortalidade específicas e padronizadas com ajuste por população em risco.

Cabe ainda destacar que a análise se restringiu à causa básica de óbito, não utilizou taxas padronizadas por falta de dados populacionais indígenas por município, e que a heterogeneidade e as limitações dos dados podem afetar a robustez das tendências observadas. A imputação de valores mínimos, em anos sem registros, foi necessária para viabilizar o ajuste do modelo Joinpoint em uma série marcada por longas sequências de zeros. Contudo, reconhece-se que essa estratégia pode aumentar a instabilidade de tendências em séries raras, motivo pelo qual a interpretação dos resultados foi realizada com cautela.

Apesar dessas limitações, confrontar os resultados obtidos neste estudo com os de investigações já realizadas contribui para melhorar a compreensão da dinâmica das mortes por doenças respiratórias infecciosas entre povos indígenas de Minas Gerais. 

Conclui-se que o número de óbitos por doenças respiratórias infecciosas entre indígenas residentes em Minas Gerais apresentou distribuição desigual no tempo e no espaço, com maior concentração no Norte do estado e impacto mais acentuado em idosos com baixa escolaridade, residentes em áreas socioeconomicamente vulneráveis. A elevada proporção de causas não especificadas evidencia fragilidades tanto no acesso à assistência qualificada quanto no registro adequado das informações de óbito.

Diante desse cenário, recomenda-se o fortalecimento da atenção à saúde nas regiões habitadas por povos originários, com ênfase na vigilância ativa, na qualificação da notificação das causas básicas de morte por meio da atuação da Vigilância do Óbito e no enfrentamento das causas evitáveis. Entre as estratégias prioritárias, destacam-se: i) o aprimoramento do treinamento de profissionais de saúde indígena para o correto preenchimento das declarações de óbito; ii) a implantação de sistemas locais de vigilância epidemiológica e de óbitos que possibilitem monitoramento em tempo quase real; iii) o reforço da atenção primária com equipes multiprofissionais itinerantes em aldeias e comunidades rurais; e iv) a criação de protocolos de integração entre municípios, estado e Secretaria Especial de Saúde Indígena (SESAI), assegurando o acompanhamento contínuo da saúde indígena (25,26,32). Dado o recorte étnico-racial adotado, considera-se ainda essencial a qualificação dos sistemas de informação voltados à população indígena, especialmente no que se refere ao adequado preenchimento da variável raça/cor e à incorporação de marcadores territoriais mais sensíveis à organização sociocultural indígena (30,32). Tais medidas podem reduzir a subnotificação, melhorar a qualidade dos dados e subsidiar políticas públicas mais eficazes e direcionadas.

As evidências reforçam ainda a necessidade da realização de estudos que integrem dados do DSEI de Minas Gerais e Espírito Santo, associados a informações provenientes de outros sistemas de informação em saúde, como o Sistema de Informação da Vigilância Epidemiológica da Gripe e o Sistema de Informações da Atenção à Saúde Indígena, por meio de técnicas de pareamento. Essa integração pode ampliar a qualidade das análises, permitindo identificar de forma mais precisa os fatores associados aos óbitos indígenas por doenças respiratórias infecciosas e subsidiar ações de vigilância e planejamento em saúde culturalmente sensíveis e territorialmente direcionadas.
